# A Review of Microwave Thermography Nondestructive Testing and Evaluation

**DOI:** 10.3390/s17051123

**Published:** 2017-05-15

**Authors:** Hong Zhang, Ruizhen Yang, Yunze He, Ali Foudazi, Liang Cheng, Guiyun Tian

**Affiliations:** 1 School of Electronic and Information Engineering, Fuqing Branch of Fujian Normal University, Fuzhou 350300, China; 2 Department of Civil and Architecture Engineering, Changsha University, Changsha 410022, China; xbaiyang@163.com; 3 College of Electrical and Information Engineering, Hunan University, Changsha 410082, China; 4 Electrical and Computer Engineering Department, Missouri University of Science and Technology, Rolla, MO 65409, USA; ali.foudazi@mst.edu; 5 School of Electrical and Electronic Engineering, Newcastle University, Newcastle upon Tyne NE1 7RU, UK; liangcheng85@gmail.com (L.C.); g.y.tian@newcastle.ac.uk (G.T.)

**Keywords:** infrared thermography, NDT, microwave thermography, volumetric heating, material

## Abstract

Microwave thermography (MWT) has many advantages including strong penetrability, selective heating, volumetric heating, significant energy savings, uniform heating, and good thermal efficiency. MWT has received growing interest due to its potential to overcome some of the limitations of microwave nondestructive testing (NDT) and thermal NDT. Moreover, during the last few decades MWT has attracted growing interest in materials assessment. In this paper, a comprehensive review of MWT techniques for materials evaluation is conducted based on a detailed literature survey. First, the basic principles of MWT are described. Different types of MWT, including microwave pulsed thermography, microwave step thermography, microwave pulsed phase thermography, and microwave lock-in thermography are defined and introduced. Then, MWT case studies are discussed. Next, comparisons with other thermography and NDT methods are conducted. Finally, the trends in MWT research are outlined, including new theoretical studies, simulations and modelling, signal processing algorithms, internal properties characterization, automatic separation and inspection systems. This work provides a summary of MWT, which can be utilized for material failures prevention and quality control.

## 1. Introduction

**Figure 1:**
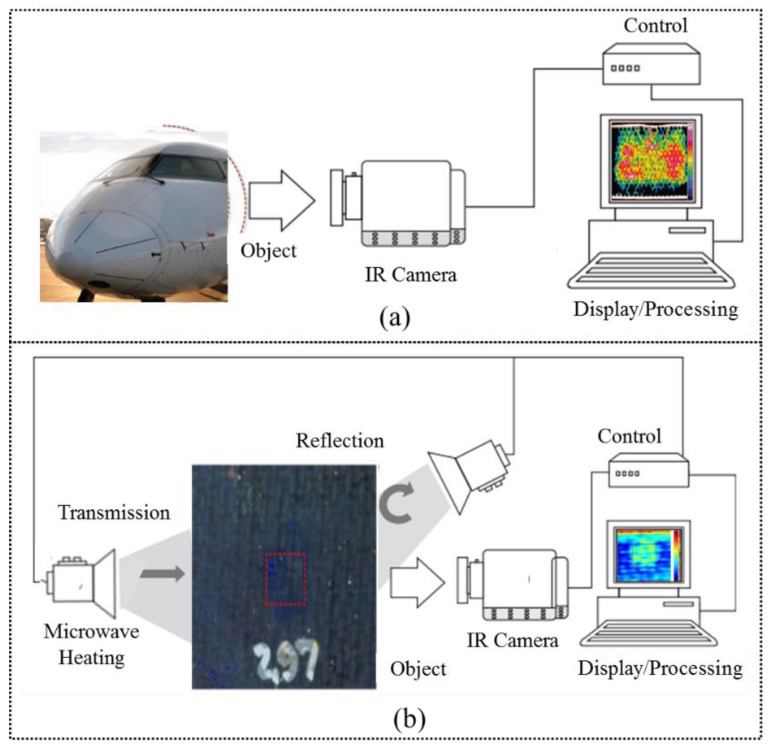
MWT setup for (**a**) the passive approach and (**b**) the active approach.

Infrared (IR) thermography plays an important role in structural health monitoring (SHM) [1] and non-destructive testing (NDT) [2]. IR thermography has great potential and advantages, including fast inspection time, high sensitivity and spatial resolution owing to commercial IR cameras’ ability to detect inner defects as a result of heat conduction. It can be split into two categories: passive and active. For the passive approach, the IR camera is used to measure the temperature of materials under test without any external excitation source. The passive thermography configuration is illustrated in Figure 1a. In many industrial processes, passive thermography has been used in production and predictive maintenance [3]. While passive thermography allows qualitative analyses to be performed, active thermography is both qualitative and quantitative [4].

Contrary to the passive approach, an external thermal excitation is required for active thermography. The known characteristics of this external excitation enable depth quantification in composites’ debonding detection [5]. As shown in Figure 1b, the configuration of the active infrared approach is similar to that of the passive approach, except for the utilization of an excitation source to generate a distinctive thermal contrast. As illustrated in Figure 1b, the IR camera is situated on the same side of the excitation source in reflection configuration. For transmission configuration, the IR camera is situated on the opposite side of the excitation source. The IR camera is synchronized with the excitation source by a control unit. A computer is required to process and display the obtained thermal images. To improve contrast and quantify defects, active thermography is often performed with advanced signal processing methods. Normally, the reflection mode is suitable for detecting defects situated near the surface, while deeper defects can be detected in the transmission mode. However, the transmission approach cannot be used in some cases where the target is inaccessible [6,7,8].

Depending on the external thermal excitation, different active thermography methods have been developed, such as pulsed thermography (PT) [9], step thermography (ST) [10] and modulated thermography (MT) or so-called lock-in thermography (LT) [11]. Finally, there is pulsed phase thermography (PPT) [12], developed by Maldague and Marinetti in 1996, which combines the advantages of PT and MT [13,14].

Various physical heating sources have been adopted as thermal stimulation sources, such as thermal lamps, lasers, ultrasound devices, and electromagnetic waves. Accordingly, laser thermography [15], ultrasonic thermography and eddy current thermography [16,17] were developed. Taking eddy current thermography as an example, it combines the advantages of IR thermography and eddy current testing, such as being fast and non-contact [18,19,20,21]. Eddy current thermography can heat many materials such as metals and carbon fiber reinforced polymer (CFRP) with eddy current heating. However, it only works for conductive materials [22,23,24], therefore, the excitation source needs to be chosen according to the specific problem. In the last decade, researchers have shown an increased interest in microwave heating techniques. Microwave heating has been exhibited advantages of rapid heat transfer (due to volumetric heating), efficiency, heating uniformity, compact equipment, and being easy to control, etc. Meanwhile, microwave heating has emerged as a powerful platform due to dielectric loss and eddy current heating with different materials under test. So far, built microwave thermography devices have shown some unique advantages such as: (1) microwaves will produce reflection, scattering, transmission at a discontinuous interface. With microwave signal reflection and scattering in the defect area, less microwave energy can be used for heating, and the temperature raise in a defect area is slower than in a non-defect area during microwave heating. IR cameras will capture this abnormal thermal image which will strengthen the effectiveness of defect detection; (2) the heating pattern of microwave heating is relatively uniform, volumetric and selective, and can be achieved in a short time; (3) microwave heating is easy to control, and it is easy to implement different heating function modulations. However, it is necessary to restrict microwave leakage as they are dangerous to human health, therefore, the leakage of the microwaves needs to be kept below a certain recommended level. Generally, in industry microwave heating is operated from 890 MHz to 2.45 GHz to minimize any possible interference with communication services [25].

Thermography has been associated with microwaves in numerous applications. MWT has been employed by some scientists to detect wet rotten wood [26], mines and surrogate signatures [27,28]. MWT has also been used to inspect and characterize various kinds of materials and phenomena, such as debonding and delamination in composite materials [29]. So far, although there are several review works [30,31,32,33,34,35,36,37,38,39,40,41,42,43,44], they have been limited to a specific field such as composite or renewable energy, so a review of MWT in the material detection field which includes the principles, advantages and disadvantages, developments and research trends is still needed. In this paper, a comprehensive review of MWT techniques for material evaluation has been provided, based on a detailed literature survey.

The overall structure of this paper includes the following: the principle of MWT is presented in Section 2. Then, typical types of MWT applications are summarized in Section 3. Section 4 reviews the development of MWT with case studies. Then, a comparison and discussion are provided in Section 5. Trends are shown in Section 6. Finally, the conclusions are outlined in Section 7.

## 2. Principle of Microwave Thermography

The principles of MWT mainly include microwave heating and 3D heat conduction. These are analyzed theoretically in the following subsections.

### 2.1. Microwave Based Heating

The heating style of microwave thermography can be divided into volume heating and surface heating [45], therefore the heating process can be divided into volumetric heating (i.e., dielectric loss heating) and surface heating (i.e., eddy current heating).

#### 2.1.1. Dielectric Loss Heating

For dielectric materials, such as glass fiber composite materials, microwave heating is volumetric heating (i.e., dielectric loss heating). Considering glass fiber composites for instance, material dielectric loss in the microwave radiation field will generate heat. The dissipated power *P* per unit volume can be expressed as follows [46]:

(1) $$P=2\pi f\varepsilon_{0}\varepsilon^{\prime \prime }E^{2}$$

where *f* is the frequency of an electric field, *E* is the RMS value of the electric field, *ε*_0_ is the permittivity of air, and *ε*” is the relative loss factor. Without considering the heat diffusion, the temperature change per unit at heating time *t* with a continuous microwave source is [46]:

(2) $$T(t)=\frac{Pt}{\rho Cp}=\frac{\omega \varepsilon_{0}\varepsilon^{\prime \prime }E^{2}}{\rho Cp}t$$

where *ρ* is the density of the material and *Cp* is heat capacity. Obviously, with constant microwave parameters and constant properties of the material under test, the temperature increases linearly with time (during a short period of time).

**Figure 2:**
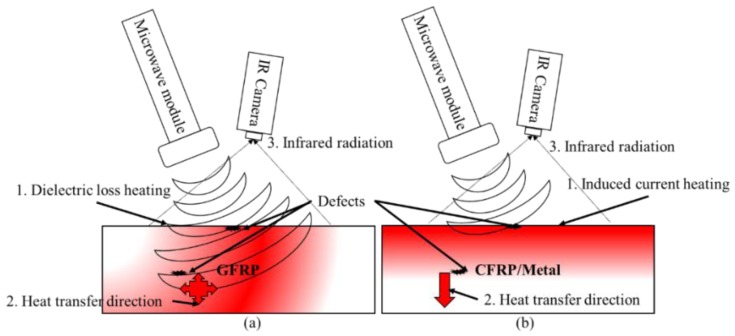
Basic schematic of MWT: dielectric material (**a**) and conductive material (**b**).

The basic principles of MWT volumetric heating are shown in Figure 2a: firstly, a microwave excitation module is used to generate a microwave radiation field; secondly, microwaves penetrate the material under test, and the medium molecules will move at the frequency of the electric field which generates heat and eventually is converted into Joule heat, which then will transfer around the material based on the diffusion equation; finally, an IR camera is utilized to obtain the temperature variation in the material under test. Due to the differences in density and heat capacity between the materials under test and defects, information about surface and internal defects can be obtained. Thus, the processes of MWT for dielectric materials evaluation are based on microwave radiation, dielectric loss to generate Joule heat, heat transfer, and IR radiation.

#### 2.1.2. Eddy Current Heating

For conductive materials, like metals and carbon fiber composites, microwave heating is eddy current heating. Since the material under test is electrically conductive, microwaves cannot penetrate the conductor material. Thus, the principle of microwave heating is that the energy is radiated to the conductive material surface by microwaves, an alternating electric field is generated, then, induced surface currents are excited from the alternating electric field, resulting in an alternating magnetic field. Next, a vortex electric field is generated by this alternating magnetic field, the vortex electric field promotes the movement of electrons which will generate Joule heat. Finally, the conduct material is heated by Joule heat as shown in Figure 2b. Power *P* and heat *Q* generated by eddy current heating can be expressed as [47]:

(3) $$P∼I_{induc\mathrm{tor}}^{2}\sqrt{\frac{\mu f}{\sigma }}$$

(4) $$Q=Pt∼I_{induc\mathrm{tor}}^{2}\sqrt{\frac{\mu f}{\sigma }}t$$

where, *I_inductor_* is the current flowing through the inductor, *σ* is the conductivity (S/m), *μ* is the magnetic permeability of the material under test and *f* is the frequency of the induced current. It is observed that with a stable induced current and induced current frequency, the generated heat is directly proportional to the microwave excitation time and it is inversely proportional to the square root of the conductivity. However, due to the presence of heat transfer and dissipation problems during actual applications, MWT must be corrected in order to minimize the measurement error.

Due to the skin effect, induced current depth (within the skin depth) in the conductive material is an extremely important factor. The skin depth can be obtained by the following equation [48]:

(5) $$\delta =\frac{1}{\sqrt{\pi \mu \sigma f}}$$

where *f* is the microwave’s frequency, *σ* is the conductivity (S/m), and *μ* is the magnetic permeability (H/m) of the material under test. A typical conductivity of CFRP is probably 1000 S/m, and the permeability is around 1. With 2.4 GHz microwave excitation, the skin depth is about 0.002 mm. Therefore, MWT belongs to the surface heating category as only the surface of the CFRP is heated.

### 2.2. 3D Heat Transfer and Temperature Field

Heat *Q* generated by dielectric loss or the Joule heat will be conducted from inside to the surrounding material. The heat conduction equation is a time-dependent heat diffusion equation [49]:

(6) $$\frac{\partial T}{\partial t}=\underset{\mathrm{Thermal}\;\mathrm{diffusion}}{\underset{︸}{\frac{k}{\rho C_{p}}\left(\frac{\partial^{2}T}{\partial x^{2}}+\frac{\partial^{2}T}{\partial y^{2}}+\frac{\partial^{2}T}{\partial z^{2}}\right)}}+\underset{\mathrm{Microwave}\;\mathrm{heating}}{\underset{︸}{\frac{1}{\rho C_{p}}Q\left(x,y,z,t\right)}}$$

where, T=T(x,y,z,t) is the temperature distribution of the surface, k is the material thermal conductivity (W/m × K), $C_{p}$ is specific heat capacity (J/kg × K), ρ is the density (kg/m^3^), and Q(x,y,z,t) is the heat generation function with microwave heating (the dielectric loss heating or eddy current heating). A surface temperature distribution will eventually reflect disturbances of the electromagnetic and thermal fields. Therefore, MWT has the potential to characterize and track the property variations of the material, such as magnetic permeability, electrical conductivity, permittivity, thermal conductivity, thermal diffusivity, etc. In addition, the depth of defects can be quantified. The heat generated by Joule heat will propagate a certain distance within the material in the form of heat waves. The penetration depth *δ_th_* of these heat waves is [50]:

(7) $$\delta_{th}\approx 2\sqrt{\alpha t}$$

(8) α=(k/ρCp)

where, *α* is the thermal diffusivity, and *t* is the observation time. *α* can be expressed as a function of the density of the material *ρ*, heat capacity *Cp* and thermal conductivity *k*, as shown in Equation (8). It shows that the penetration depth of heat *δ_th_* is proportional to the square root of *t* and *α* [11]. In the case of a modulated thermal wave, the length of thermal diffusion decides the penetration depth, which can be found from the following equation [11]:

(9) $$\mu_{t}=\sqrt{\frac{2k}{\omega \rho Cp}}=\sqrt{\frac{\alpha }{\pi f}}$$

where, *ρ* is density, *k* is thermal conductivity, *α* is thermal diffusivity, *Cp* is heat capacity, and *f* is the frequency of the thermal wave. The penetration depth is proportional to the reciprocal of the square root of *f* and *α*. In other words, the detection depth varies according to the modulation frequency. In summary, the detection ability of microwave thermography is closely related to the electrical, dielectric and thermal properties of the material under test.

## 3. Types of Microwave Thermography

### 3.1. Classification of Excitation Configuration

**Figure 3:**
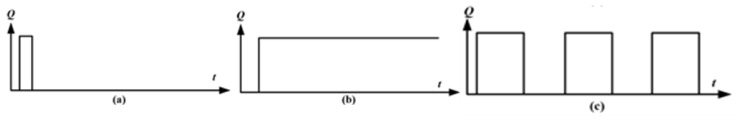
Excitation functions of MWT: (**a**) MPT; (**b**) MST; (**c**) MLT.

According to the excitation configurations of microwave heating, MWT can be divided into microwave pulsed thermography (MPT),microwave pulsed phase thermography (MPPT), microwave lock-in thermography (MLT) [51], or microwave step thermography (MST), also known as microwave time-resolved thermography [52]. With MPT, the material under test is heated by a small period of microwave excitation as shown in Figure 3a. The variation of temperature is observed in the heating phase and the cooling phase. For MST, the sample is step heated by a long pulse as shown in Figure 3b, and the variation of temperature is observed in the heating phase. As shown in Figure 3c, the material under test is heated by a periodic amplitude modulated microwave with MLT and the periodic temperature change is captured. A square pulsed modulated excitation is used to derive phase information from multiple thermal waves with one inspection. The influence of non-uniform heating is been reduced and deeper defects can be displayed with a higher contrast. A pulse excitation signal is used by MPPT. Phase analysis is carried out in the frequency domain [13]. Predictably, microwave frequency modulated thermography will employ a frequency modulated microwave excitation in order to derive phase information. From the above analysis, the conclusion can be reached that: MPT and MST analyze the temperature of thermal imaging in the time domain, which is affected by surface emissivity variations and non-uniform heating; MLT obtains information in the frequency domain such as phase, which can suppress the influence of the surface emissivity variations and non-uniform heating. However, the MLT inspection system requires a long measurement time and it is relatively complex.

Comparisons among MPT, MST, MLT, and MPPT are listed in Table 1. Due to the use of an IR camera, all of them exhibit high sensitivity, high resolution, full-field detection and good visibility. In addition, quantification information can be achieved based on the heat conduction:

1. MPT can be fast and easily deployed. Surface temperature gradients will be introduced not only from defects, but also local variations in surface emissivity and non-uniform heating. A long inspection time is required for a thick material. In addition, the material could be damaged due to the high heating energy.
2. MST is a time-resolved method and it can be used to quantify defect depth. However, the radiation from the heat source in the continuous heating process could deteriorate the temperature measurements. Also, the non-uniform heating and surface emissivity variation have adverse effects on defect evaluation.
3. MLT generally required less excitation energy than MPT. MLT exhibits a higher sensitivity than MPT. The phase data can be extracted which is independent of surface emissivity and heating variations. Tests are repeated with various frequencies and it becomes a time-consuming process to detect defects with various depths. However, MLT offer a compromise with a better depth resolution.
4. MPPT combines the advantages of MPT and MLT. MPPT is less sensitive to non-uniform heating and surface emissivity than MLT as only phase information can be obtained. Moreover, MPPT employed a short excitation pulse which is faster than MLT and wide frequency spectra can be obtained. However, with increasing frequency, the transferred energy is decreased with MPPT. With post processing algorithm, MPPT exhibits better detect ability and resolution than MPT for deeper defects.

### 3.2. Classification by Heating Style

**Figure 4:**
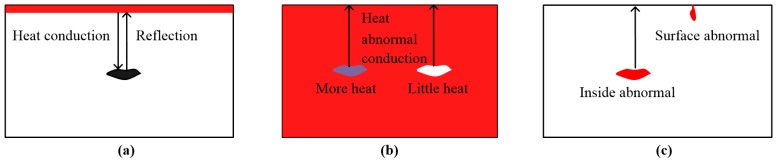
Styles of heating: (**a**) SHT; (**b**) VHT; (**c**) AHT.

MWT can be classified into surface heating thermography, volume heating thermography, and abnormal heating thermography [14,45]. In Figure 4a, for eddy current heating, MWT can be also called surface heating thermography (SHT). Due to the great permeability, the skin depth is very small [53,54,55]. Thus, it can be classified as part of the SHT family. With reflection mode, the heat conduction from surface to inside is used to quantify the depth of defect.

MWT exhibits volumetric heating for dielectric material inspection. MWT can be called volume heating thermography (VHT), as illustrated in Figure 4b [45,55]. For the transmission and reflection modes with VHT, the characterizations of defects are similar [54]. Only interesting areas are heated without heating the host material in some cases. Abnormal heat will caused by defects. Furthermore, this abnormal heat is used to quantify depth information of the related defect. In Figure 4c, these methods are called abnormal heating thermography (AHT). For detecting water in concrete structures, MWT can be considered as a kind of AHT.

## 4. Developments and Case Studies

### 4.1. A History of MWT Development 

Developed countries have taken the lead on the use of MWT to carry out related researches and achieved some interesting results [56,57,58]. Levesque and Ambrosio did a preliminary study on MWT in the 1990s. Levesque et al. employed X and Ku bands (range from 8 GHz to 18 GHz) horns and parabolic antennas to excite the sample under test [59]. The excitation of the antenna is provided by a 100 W amplifier. Thermal images are produced by an AGEMA (model 782 LWB) IR camera with 8 μm–12 μm range. An area of 300 × 300 mm^2^ is been measured due to the limited excitation area. Several 10 mm thick glass-epoxy composites have been tested to identify the inserted carbon particles area. The proposed method were used to characterize artificial defects’ size and location under different depths in composite materials. Ambrosio et al. used a cavity with a minimum 600 W to detect artificial defects in non-metallic composites [60]. The cavity was 300 × 200 × 280 mm^3^ which is excited by a 2.45 GHz magnetron and an Alter VPG1540 power supply unit (maximum power 1200 W). They used a Cober PM45 power meter to monitor the incident and reflected microwave powers. Two cavity applicators (an open cavity applicator and a large cavity applicator) have been proposed to avoid the aperture edge effects and to improve the field uniformity over the sample. To estimate the defect permittivity and depth influence on surface temperature perturbation, theoretical modeling and numerical simulations were carried out.

In 1999, Takahide et al. at Osaka University applied MWT to detect surface breaking cracks in reinforced concrete structures and they pointed out that the tip of crack will produce more heat during the test [61]. Heat was introduced by a time-gated microwave source into the homogeneously reinforced concrete structure. With the microwave penetration features, the subsurface of the reinforced concrete structure could be inspected. Moreover, wet cracks could be selectively heated. Therefore, subsurface cracks will be immediately identified with MWT. Analyses of time and spatial dependence features of measured thermal images are possible to quantify cracks’ depth and thermal diffusivity information [61]. In the 21st century, groups in the USA, UK, France, Poland, Italy, Korea, etc. have developed MWT for metal, dielectric material, cement/concrete, GFRP, CFRP, honeycomb and cement-based composites detection problems. These works have been simply summarized in Table 2 and are introduced in detail in the following Section 4.2, Section 4.3, Section 4.4, Section 4.5, Section 4.6, Section 4.7, Section 4.8 and Section 4.9.

### 4.2. Metals and Corrosion

**Figure 5:**
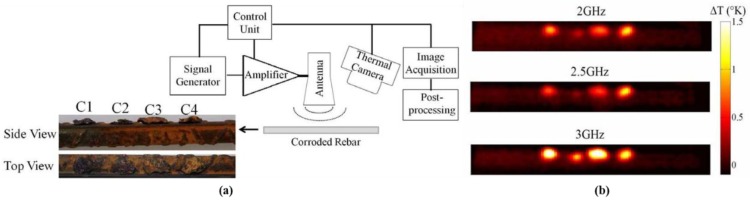
(**a**) MWT measurement setup for corroded rebar detection; (**b**) temperature profile for corroded steel bar [62]. Reprinted/reproduced with permission from IEEE. .

Foudazi et al. from the Missouri University of Science and Technology proposed the use of MWT for the detection and characterization of corroded reinforced steel bars [62]. In Figure 5a, the measurement setup for steel bars is illustrated. They employed a 14 × 24 cm^2^ horn antenna to irradiate steel bars with a 50 W microwave signal for 10 s. The distance between the steel bars and the antenna was 1 cm. During the experiment, four pieces of corroded material were mounted on steel bars and spaced 1 cm apart. A DRS Tamarisk 320 thermal camera was utilized to obtain the thermal profile of the steel bars with a 0.05 K sensitivity. In Figure 5b, the surface thermal profiles for steel bars after 10 s as detected with 2 GHz, 2.5 GHz, and 3 GHz energy are provided. The steel bars were a smooth rebar without ribs. The radius of this rebar was 4.8 cm which contained a 1 cm corrosion area. In Figure 5b, the visible hot spots are the corroded areas on these steel bars. Because of the relatively low thermal conductivity of corroded steel, heat quickly dissipates in uncorroded steel. Moreover, these temperature differences between the corroded areas indicated that different amounts of corrosion absorb different amounts of microwave energy. With a preliminary simulation and experimental study of the MWT, it demonstrated that a higher excitation microwave frequency will lead to a higher temperature for corrosion detection in steel bars. Moreover, increased corrosion leads to absorption of microwave energy increasing results in a greater difference in the measured IR images.

Pieper et al. demonstrated an active MWT for inspection of large corrosion areas on reinforcing steel bars for cement-based structures [63]. They used CST Microwave studio and MultiPhysics studio to construct a coupled microwave and thermal simulation model. The effects of steel bars have been investigated in the air and in concrete. To detect the change in temperature with a thermal camera, the incident microwave power should be increased because some of the microwave energy is absorbed by the concrete. The effect of different polarization has been studied with CST. With 10 W incident power, the parallel polarization generates the most uniform heating in that of orthogonal polarization and circular polarization, but the temperature increase was the lowest (only 0.014 °C). The highest temperature increase was generated by orthogonal polarization (around 0.025 °C), therefore, orthogonal polarization is the best choice for thin corrosion detection.

**Figure 6:**
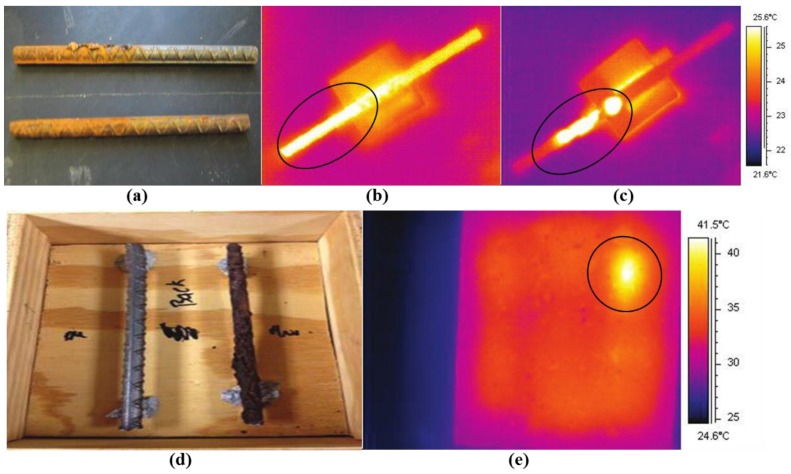
(**a**) Photograph of localized corrosion (top) and light corrosion (bottom); (**b**) thermal images of a steel bar with light corrosion and (**c**) localized corrosion; (**d**) photograph of clean (left) and corroded (right) steel bars; (**e**) thermal images of clean (left) and corroded (right) steel bars embedded in the concrete block after microwave heating [63]. Reprinted/Reproduced with permission from AIP Publishing LLC.

During the experimental study, two steel (AISI 1008) bars (each of length 150 mm and radius 4.8 mm) were measured, which have been embedded in parallel in a concrete block (170 × 150 × 50 mm^3^). The sample was heated for 5 s by a microwave oven operating at 2.45 GHz. A FLIR Thermacam SC 500 was used to provide thermal images with a sensitivity of 0.1 °C. In Figure 6a, the two steel bars have been heated by microwave energy for 15 s. One (top) with localized significant corrosion (on the order of 1 mm–4 mm) on a portion of its length, the one below with light corrosion (on the order 0.2 mm or less) along half of its length. As illustrated in Figure 6b,c, the corroded areas in the two steel bars have been identified in the thermal images as indicated by the black circle. Moreover, in Figure 6c, the uncorroded area in the steel bar is also visible, appearing as a relatively lower temperature line (in the left of the black circle). In Figure 6e, the corroded steel bar is still detectable when embedded in a concrete block as highlighted by the black circle. These results show that steel corrosion in concrete will lead to a higher loss tangent and the thermal properties are changed. With microwave heating, the physical property changes due to corrosion become measurable in the thermograms. Depending on the orientation and thickness of corrosion, the polarization of the incident microwave signal can be optimized through simulation. The effect of corrosion layer thickness on microwave heating has been investigated.

**Figure 7:**
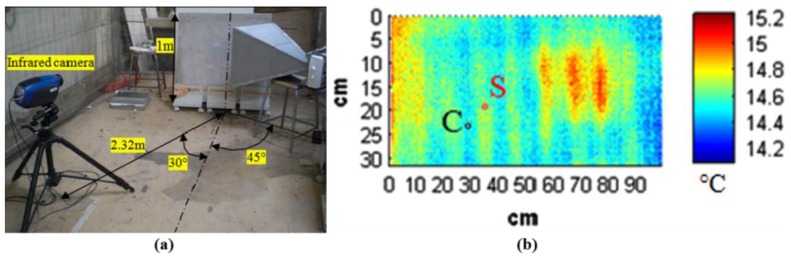
Photograph of microwave thermography measurement setup (**a**) and temperature profile for corroded steel bar (**b**) [65]. Reprinted/reproduced with permission from Elsevier.

Keo et al. presented a pyramidal horn antenna based MWT to detect corroded steel in a reinforced concrete wall [65]. During the experimental study, a commercial magnetron operating at 2.45 GHz was used to irradiate a maximum 800 W of microwave energy. The steel reinforcements (each one with a diameter of 12 mm, located at a regular spacing of 10 cm) with a 38 mm concrete covering were embedded in a concrete block (1000 × 1000 × 65 mm^3^). The specimen was heated by 600 W microwave energy for 5 min. The reflection mode was used as the thermal camera was placed on the same side as the excitation source. A data analysis method based on a contrast algorithm was used to analyze the thermogram series to reduce the effect of non-uniform excitation. As shown in Figure 7a, the infrared camera was composed of a 320 × 256 matrix detector of indium antimonide (InSb) with a sensitivity range of 3–5 μm. The excitation antenna was placed in a 45° direction to heat the largest surface area of the specimen. In addition, the infrared camera was placed at 2.32 m away from the specimen in a 30° direction which can detect the largest possible surface area of the specimen. Thermograms were recorded at 1 image per s by using the ALTAIR software (developed by FLIR for obtaining measurement data). Figure 7b shows the obtained thermograms at the 250 s instant. The abnormal temperature areas correspond to the corrosion area in the steel reinforcements. In the preliminary experimental study of the MWT, it was demonstrated that the detection depth of MWT was about 3.8 cm in a thick concrete block.

### 4.3. Dielectric Materials

**Figure 8:**
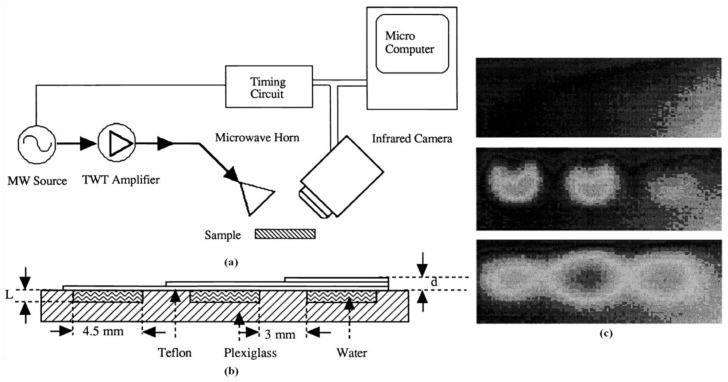
Schematic diagram of the experimental setup (**a**), the specimen under test (**b**) and IR images (**c**) [66]. Reprinted/reproduced with permission from SPIE.

Osiander et al. employed a time-resolved MWT system for surface and sub-surface inspection on Plexiglass-water-Teflon specimens with absorbers at different depths (0.15, 0.30 and 0.45 mm) [66]. Figure 8a shows a diagram of the experimental setup. An HP 6890B oscillator was used to generate microwave pulses with a frequency range from 5 GHz to 10 GHz. A Hughes 1277 X-band traveling wave tube amplifier with 2.3 W maximum power was utilized as a power amplifier. An excitation horn antenna was placed in a 45° direction with respect to the sample surface. Two infrared cameras were used to monitor the surface temperature of the sample under test. One was a Mikron 6T61 infrared scanner with an HgCdTe detector. The second one was a SantaBarbara infrared camera with a 128 × 128 pixels InSb focal plane array. The temperature resolution of the first camera was 0.1 K. For the second IR camera, the temperature resolution is about 0.003 K. The specimen under test is illustrated in Figure 8b. It is structured with a Teflon layer of varied thickness and a water layer with a constant thickness backed with Plexiglass layers. Considering the power, the microwave horn was working in the near field. The measured data was normalized to the peak amplitude to eliminate the effects of non-uniform microwave distribution. Figure 8c shows IR images of the test sample before, during and after a 2.7 s microwave pulse. Three water layers corresponding to the Teflon layer thicknesses appear in the IR images in a temporal order. With MWT, the potential of surface temperature versus time has been shown for subsurface defects quantitative characterization. Compared with laser beam heating, dry epoxy coated steel samples exhibit a very small response with microwave heating. When a debonding region contains water, the whole structure of the debonding region can be illustrated. The wavelength independent resolution has been demonstrated to be 30 μm. An analytical model has been provided to extract the time dependence of the surface temperature where quantitative data such as the depth of the defect can be extracted.

### 4.4. Cement and Concrete

**Figure 9:**
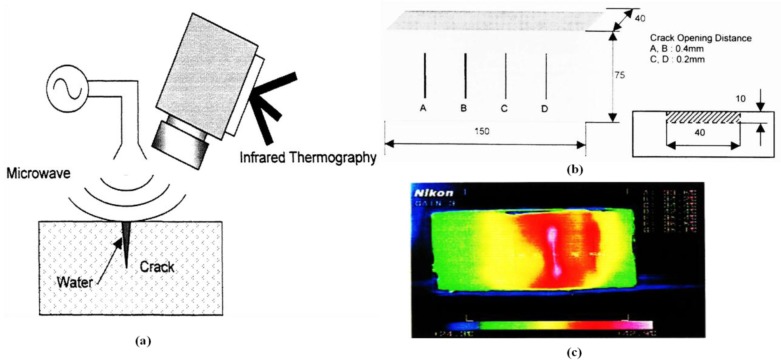
Schematic diagram of MWT (**a**), mortar block specimen with artificial cracks (**b**) and thermal images taken after microwave heating (**c**) [61]. Reprinted/reproduced with permission from SPIE.

Takahide et al. proposed the use of MWT for detecting surface-breaking cracks in concrete [61]. Surface-breaking cracks can be penetrated with water. The crack opening distance was 0.2 mm and 0.4 mm. The size of the cracks was 10 mm in depth and 40 mm in width. Since the microwave absorptivity of the concrete is much smaller than that of water, cracks containing water can be selectively heated. By applying microwaves to the concrete structure, the thermal conduction of these heated cracks will generate localized high-temperature regions. The experimental MWT setup is illustrated in Figure 9a. A commercially available 2.45 GHz magnetron was used to irradiate microwaves into the mortar block with a maximum 1400 W output power. The specimen under test was placed in the microwave oven. A Nikon LAIRD-3 with PtSi array was used to measure the temperature distribution of the mortar block. In Figure 9b, four artificial cracks have been introduced to a rapid hardening mortar block specimen. By injecting water into crack C and applying microwaves for 5 s, crack C with water was selectively heated. Compared with the non-crack area, the temperature of the crack rose about 6 °C to 8 °C after microwave heating. As shown in Figure 9c, the position of the crack C can be easily detected from the thermal image taken immediately after the microwave excitation. Meanwhile, the authors found that the temperature region of the crack degraded after 20 s due to the thermal diffusion from the crack into the surrounding area. The relation between the crack size and temperature rise should be determined for quantitative investigations.

### 4.5. Glass Fiber Composite Structures

Bowen et al. introduced microwave-source time-resolved infrared radiometry for detecting and characterizing microwave absorption in dielectric materials [67]. An HP 6890B oscillator was used to excite a 9 GHz microwave signal. A Hughes 1277 X-band traveling wave tube amplifier was used to amplify this signal to a maximum 2.3 W power. A single flare horn antenna with about 50° beam width was placed 15 cm from the sample under test. A Santa Barbara camera with 128 × 128 InSb focal plane array was used to detect the IR radiation. The temperature resolution is about 0.003 K. With embedded fibers at different locations, a fiberglass-epoxy specimen was measured. The depth of the fibers was 0.25 mm and 0.75 mm. Due to the loss tangent and Joule heating, there is a very high contrast for defects in embedded epoxy materials. The size and orientation of these embedded fibers in the measured thermal images have been studied. The interaction is strongly dependent on the length of the fiber (9 mm to 30 mm) and orientations. The temperature shows a modal distribution along the fiber for fibers longer than 12 mm.

**Figure 10:**
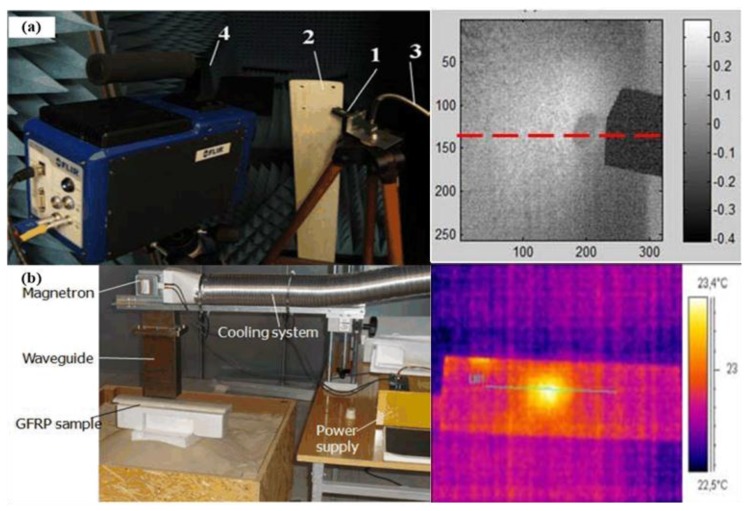
MWT setup in Newcastle University (**a**) and West Pomeranian University of Technology (**b**) [57]. Reprinted/reproduced with permission from IEEE.

Cheng et al. [57] developed a microwave pulsed thermography system for glass fiber composite measurement. Figure 10a illustrates the setup of the experimental system: (1) an adaptor connected with the waveguide; (2) GFRP wind turbine blade; (3) microwave generator linked with cable; (4) IR camera. GFRP wind turbine blade with 4 holes (4.5 mm in radius) at the root was heated by an open-ended waveguide connected with a Flann 18 094-SF40 adaptor. The adaptor was linked to a signal generator (Rohde & Schwarz SMF 100 A) with the maximum output power of 30 dBm (1 W) at 18 GHz. The waveguide was placed above the sample with roughly a 45 degree illumination direction. A FLIR SC7500 IR camera was used to obtain the temperature distribution. Artificial holes with 4.5 mm radius were investigated. During the experiment, the signal generator provided 1 W microwave power, although only 115.2 mW microwave power was emitted from the waveguide due to the cable loss. In order to maximize the actual illumination power and minimize the power loss during the transmission, 22.7 mm was selected as the standoff distance (distance between the microwave antenna aperture and sample under test) due to the impedance matching (this distance should be around λ/4 to maximum power transfer, where λ is the wavelength of microwave). In their primary experimental results, a discontinuous temperature was captured in the defect region (mainly at the edges). A high power heating system is required for a better contrast of heat patterns between defect and non-defect regions.

A magnetron-based microwave pulsed thermography system has been developed by West Pomeranian University of Technology for glass fiber composite debonding and delamination detection [57]. A GFRP wind turbine blade (BLADES200W from Navitron) with a 4.5 mm hole at the middle was the study object. Figure 10b illustrates system setup for high power microwave pulsed thermography. With a maximum 1 kW power, the microwave excitation was a 2.45 GHz magnetron. An open-ended aperture was matched impedance with a WR-430 waveguide. Dipolar Magdrive 1000 was used to control the output power. A Flir A325 device was used to obtain thermograms. A 0.238 °C maximum temperature raise can be obtained from 2 s heating. In their primary experiment results, the location and the size information of the defect have been detected from the continuities in the line-scan results.

**Figure 11:**
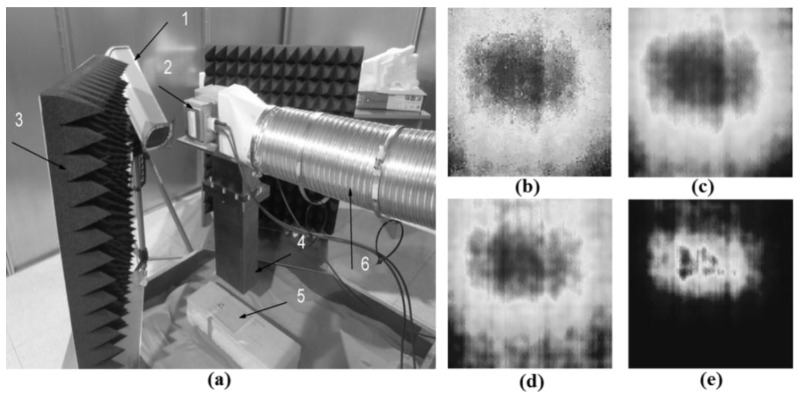
Experimental setup for MWT (**a**) and measurement results (**b**–**e**) [68]. Reprinted/reproduced with permission from authors.

Ryszard et al. also employed MWT for inspecting adhesive joints in composite materials [68]. Due to the dielectric property differences between defects (lack of adhesive or delamination) and the background, the induced thermal energy in the defect region and the background in the material under test is different. The experimental setup is illustrated in Figure 11a: (1) an IR camera in a protective box to prevent damage caused by the high power microwaves; (2) a 500 W magnetron working at 2.45 GHz; (3) microwave absorbers; (4) an open-ended rectangular waveguide; (5) the sample under test; (6) a cooling system for the magnetron. The MWT observation time was set to 180 s to obtain 180 thermograms in one sequence. The measured results were obtained by using dedicated image processing algorithms. As shown in Figure 11b–e, the original thermogram (b), cosine transform (c), standardized contrast (d) and contrast enhancement (e) were used to obtain images of the defect. In the primary experiment results, approximate location and the size information about delamination can be obtained in the thermograms after processing.

**Figure 12:**
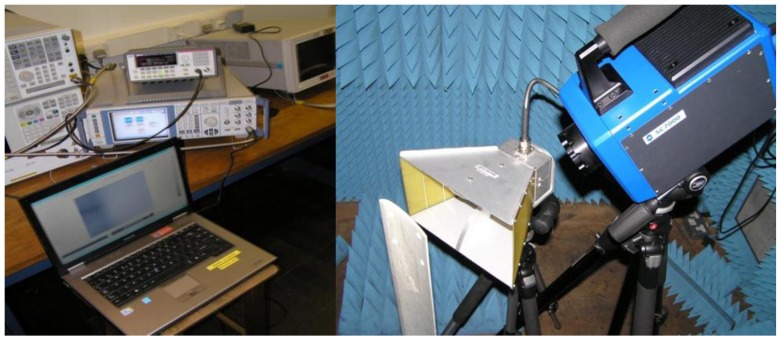
Experimental setup for defect detection with MWT.

**Figure 13:**
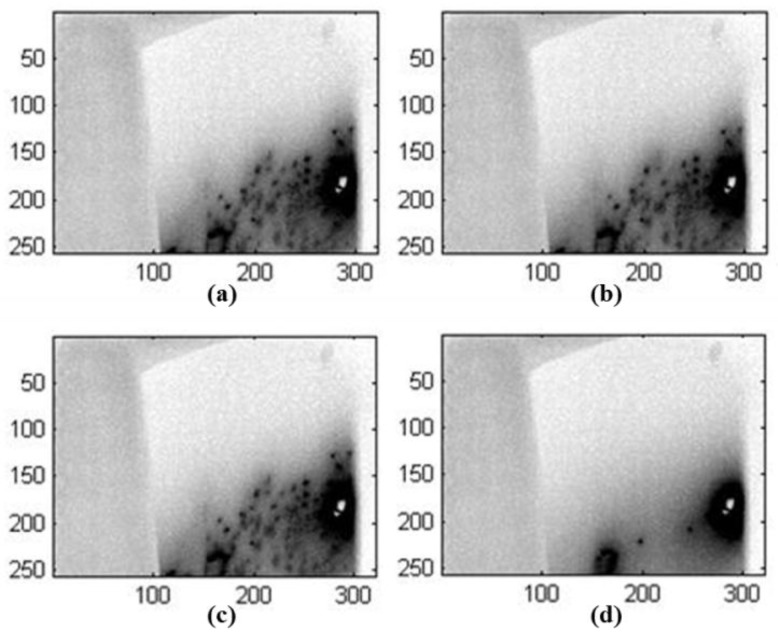
IR results with MWT for defect detection in glass fiber wind blade after 1 min microwave excitation with 0.915 GHz (**a**), after 2 min microwave excitation with 0.915 GHz (**b**), after 1 min microwave excitation with 2.45 GHz (**c**) and after 2 min microwave excitation with 2.45 GHz (**d**).

We also investigated MWT for defect detection in a glass fiber wind turbine blade, as shown in Figure 12. A horn antenna (ETS Lindgren 3115, frequency range: 750 MHz–18 GHz.) was used for microwave excitation. The antenna was connected to Rohde & Schwarz SMF 100 A signal generator with a maximum output power of 1 W. The waveguide was placed above the sample with roughly 90 degree illumination direction. A FLIR SC7500 IR camera was used to obtain the temperature distribution of sample under test. During the experiment, the signal generator provided 1 W at 0.915 GHz and 2.45 GHz. In order to detect defects during the experiment, the microwave heating duration was selected as 1 min (as shown in Figure 13a,c) and 2 min (as shown in Figure 13b,d). In our initial experiment results, a discontinuous temperature was captured in the defect region. Furthermore, the heating effect of 2.4 GHz is not as good as 0.915 GHz due to the penetration ability difference. With the captured IR images, defects around 1 mm radius with 0.2 mm in depth could be obtained in the GFRP sample. With a longer heating duration, the number of defects became hard to identify due to the heat diffusion, therefore, the heating time must be optimized for different situations.

### 4.6. Carbon Fiber Composite Materials

**Figure 14:**
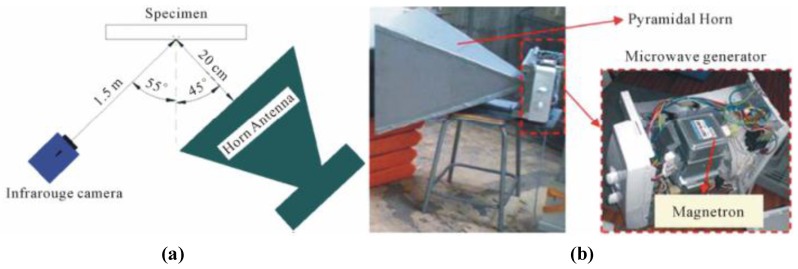
MWT setup (**a**) and Horn (**b**) in University Institute of Technology of Bethune [56]. Reprinted/reproduced with permission from Scientific Research Publishing Inc.

**Figure 15:**
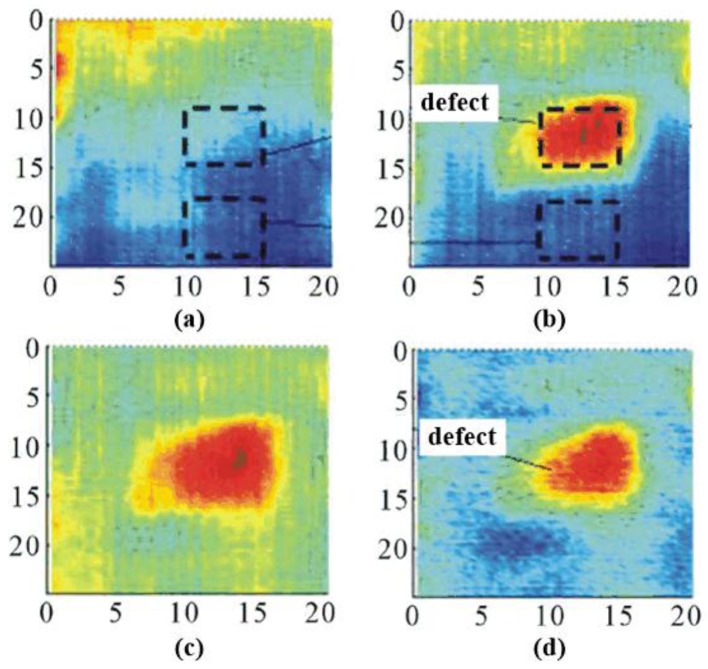
MWT results for the absence of adhesive in CFRP: the thermogram of the sample without defect (**a**), the thermogram of the sample with the defect (**b**), the subtraction between the thermograms of the sample with and without the defect (**c**), thermogram subtracted from its initial thermogram (**d**) [56]. Reprinted/reproduced with permission from Scientific Research Publishing Inc.

In France, Keo et al. developed a microwave pulsed thermography for CFRP detection [56]. They used a commercial 2.45 GHz magnetron to generate microwave signals. With a detector with a 320 × 256 InSb matrix, the sensitivity of the IR camera is in a range of 3 μm–5 μm. To capture the whole inspection area, the sample was placed 1.5 m away from IR camera at 55°. The CFRP sample was 40 × 40 × 4.5 cm^3^. A 10 × 10 cm^2^ defect was created at the middle of the CFRP sample by the absence of adhesive. The antenna was placed in the 45° direction as shown in Figure 14a. In Figure 14b, the microwave beam was guided by a 59 × 56 cm^2^ pyramidal horn antenna onto the sample under test. With the ALTAIR program provided by FLIR, a computer was used to record thermograms at 1 Hz. A 360 W microwave was used to heat the specimen for 150 s. The same testing procedure was carried out on samples with/without defect. As shown in Figure 15, the thermograms at the 100 s were obtained. In Figure 15a, a non-defect specimen has been shown in the thermogram at the 100 s instant. In Figure 15b, a specimen with a defect has been shown in the thermogram. In Figure 15c, the thermogram of the sample with a defect (Figure 15a) has been subtracted with the thermogram of the sample without defect (Figure 15b) at the same instant. In Figure 15d, the thermogram in Figure 15c is subtracted from its initial thermogram in order to highlight the defect. In the primary experiment results, the CFRP defect area can be clearly found. It is hotter than the non-defect area due to the absence of adhesive. The defect area can be estimated directly from the thermograms (about 10 × 10 cm^2^) [56].

**Figure 16:**
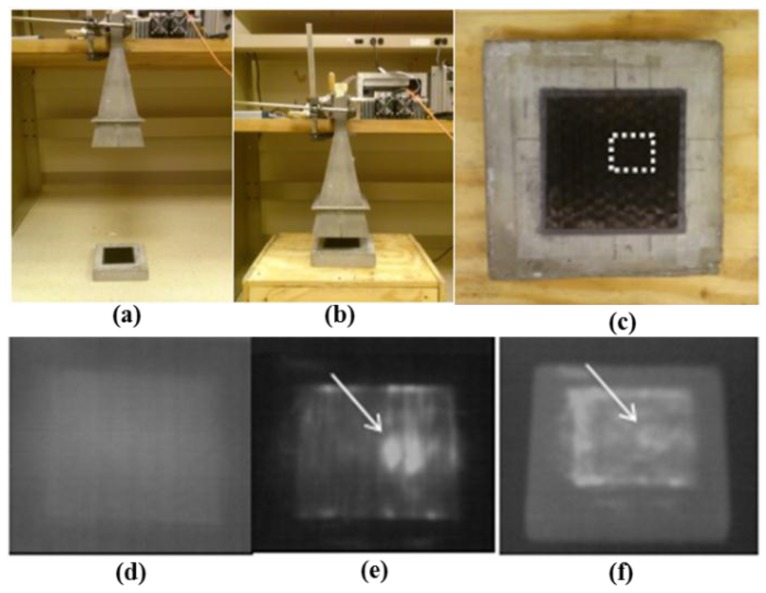
MWT measurement system setup used for 45 cm (**a**) and 6 cm (**b**) standoff, photograph of mortar sample with delamination (**c**). Measurements on mortar sample before heating (**d**), 6 cm standoff after 5 s heat (**e**) and 45 cm standoff after 15 s heat (**f**) [58]. Reprinted/reproduced with permission from IEEE.

In 2014, Foudazi et al. investigated MWT for the inspection of rehabilitated cement-based structures [64]. The experimental setup is shown in Figure 16a,b, 2.4 GHz microwaves have been transmitted with 50 W power. A sweep oscillator (HP8690B) and a power amplifier (OphirRF 5303084) are used to generate high power microwave signals. The sample under test was illuminated by a horn antenna. With a sensitivity of 0.05 K, the thermal profile of the sample’s surface was captured by an IR camera (DRS Tamarisk 320). In Figure 16c, the sample is a 20 × 20 × 4 cm^3^ mortar sample covered with a 13 × 13 cm^2^ CFRP. There is a 2 × 2 cm^2^ and 2 mm depth delamination in the center of the sample [58]. With 5 s heating time, measurements were performed at a 6 cm standoff distance. Meanwhile, measurements were also performed at a 45 cm standoff distance with 15 s heating time. Thermal profiles were captured before and after heating. In Figure 16d, the thermal image before heating is provided. Figure 16e shows the thermal image after 5 s heating. In their primary experiment results, the delamination in CFRP can be easily identified. Both the level of power and heating time should be optimized to detect small size disbands. In addition, they found that the measured result is evidently affected by edge effects with 45 cm standoff distance, as shown in Figure 16f.

**Figure 17:**
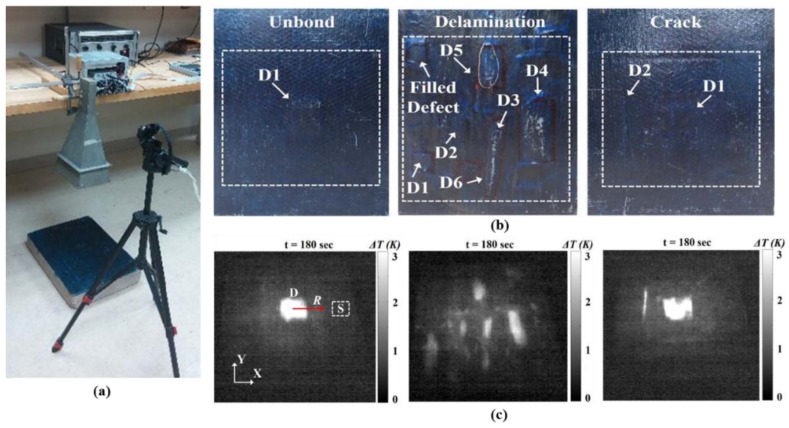
MWT measurement setup (**a**), samples under test (**b**) and thermal profiles for samples under test (**c**) [70]. Reprinted/reproduced with permission from IEEE.

Recently, Foudazi has shown that the MWT method can be used for detection of delaminations, debonding and cracks in rehabilitated cement-based materials [70]. The experimental setup is shown in Figure 17a. Three CFRP-strengthened cement-based specimens have been measured (a unbonded defect was made by placing a 5 mm thin sheet of foam with dimensions of 6 cm × 8 cm in a sample with 52 × 38 × 9 cm^3^, several delaminations (with thicknesses ranging from 1 mm to 3 mm and areas ranging from 10 cm^2^ to 100 cm^2^) have been formed in a sample with dimensions of 52 × 38 × 7.8 cm^3^, the crack sample had dimensions of 52 × 38 × 9 cm^3^), as shown in Figure 17b. Thermal profiles for these specimens are illustrated in Figure 17c. The approximate location and the size information about defects in these specimens have been obtained. Meanwhile, the temperature of the defect will be affected by the orientation of the carbon fibers (due to the electric field difference). In their initial experimental results, for the case of unidirectional carbon fiber, if the electric field is along the fiber direction, the temperature change for the defected area will be smaller compared to the case of perpendicular polarization. This is due to the different electromagnetic response of the carbon fiber at different polarizations. In other words, if the wave is parallel to the fiber orientation, it will react as an electric conductor, while for the perpendicular case it is similar to high loss dielectric materials. Additionally, the thermal contrast (TC) between healthy and defective areas is much greater. Moreover, increasing defect dimensions also led to a greater TC.

**Figure 18:**
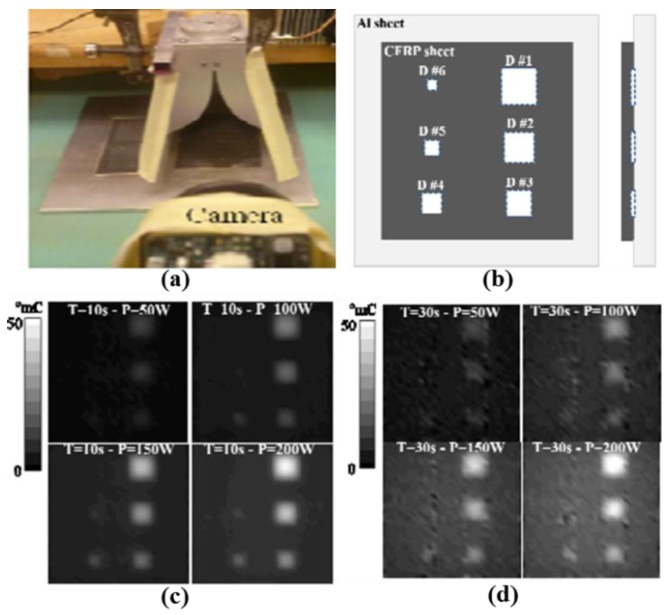
MWT experimental setup (**a**), sample (**b**), measurement results for 10 s heating (**c**) and 30 s heating (**d**) [64]. Reprinted/reproduced with permission from IEEE.

Furthermore, the authors used MWT to monitor debonding in CFRP by using different excitation antennas [64]. A horn antenna linked with a double-ridged waveguide operating at frequency ranged from 0.75 GHz to 18 GHz was employed. The aperture size of this antenna is 14 × 24 cm^2^. In Figure 18a, the CFRP sample is 30 × 30 × 0.2 cm^3^ backed with 40 × 40 × 5 cm^3^ Al sheet which containing six debonds with dimensions of 6 × 6 cm^2^, 5 × 5 cm^2^, 4 × 4 cm^2^, 3 × 3 cm^2^, 2 × 2 cm^2^, and 1 × 1 cm^2^, respectively. Figure 18b shows top and side views of the CFRP backed by a layer of Al sheet. During experiment, the sample under test was illuminated at 2.4 GHz microwave signal of different power (50, 100, 150 and 200 W). In addition, the effect of the heating time (from 10 s to 30 s) has been investigated by using CST Microwave Studio and MultiPhysics Studio. As shown in Figure 18c,d, normalized thermal images of CFRP with different excitation power levels at 10 s and 30 s have been demonstrated. In the preliminary experimental results, they found that a higher excitation microwave power is needed for a smaller debond detection. In addition, the debonding becomes clearly visible with increasing excitation power due to the temperature difference increase. Therefore, increasing the incident power improves the detection of small disbonds. Meanwhile, an increase of the heating time leads to an increased temperature throughout the sample under test, thereby the possibility of detecting the disbonds is reduced, therefore, the heating time must be optimized according to the actual situation.

**Figure 19:**
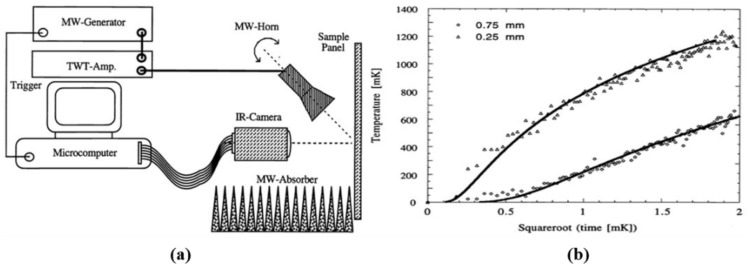
Schematic diagram of the experimental setup in The John Hopkins University (**a**) and Surface temperature as a function of square root time for a single point on fibers in 0.25 mm and 0.75 mm depth (**b**) [71]. Reprinted/reproduced with permission from SPIE.

A microwave time-resolved infrared radiometry system was proposed by scientists in The John Hopkins University [71]. The experimental setup is shown in Figure 19a. 9 GHz microwave signals were produced by an HP 6890B Oscillator. A Hughes 1277 X-band traveling wave tube amplifier was used to amplify these signals and then to feed them into a rectangular waveguide linked with a single flare horn antenna. The beam width of this horn antenna is about 50° and the sample was placed about 15 cm away. 2.3 W input power was transmitted to the antenna and a 20 mW/cm^2^ power density was created for the experimental study. Both the polarization of the microwave and the angle of incidence were controlled. By operating in the 3 μm–5 μm band, a 128 × 128 InSb focal plane array infrared camera was used to monitor the surface temperature of the sample under test. Before, during and after the application of the microwave step heating pulse, a series of frames were recorded for time-dependent measurements. In the initial experimental results, the authors measured carbon fibers with two different depths (0.25 mm and 0.75 mm) in fiberglass-epoxy. The temperature at the center of the fiber is shown in Figure 19b. Moreover, they found that the experimental data can be fitted with a solid line. The determination of surface layer thickness and thermal diffusivity can be almost independent from the surface properties of the layer.

Lee et al. proposed a microwave probe pumping technique to characterize the anisotropic electrical conductivity in carbon-fiber composite materials [69]. They used CST Microwave Studio to investigate the electromagnetic field distribution with different anisotropic conductivity. Two 10 mm coaxial probes were used to pump and scan the microwave field. The pumping probe was fixed on the backside of the sample under test. The near-field distribution was scanned by the scanning probe. A spectrometer was used to measure the microwave power. A network analyzer was used as the microwave source with a continuous mode. A FLIR T620 was used for measurement. A 1 GHz microwave source with 20 dbm power was used during the measurements. A 50 × 50 × 0.1 mm^3^ carbon-fiber/PEEK composite sheet with a defect (characteristic length is 5 mm) has been measured. In the initial experimental results, they obtained an intense area around the scanning probe. Moreover, they found that the conductivity of the carbon-fiber/PEEK has an elliptical distribution.

### 4.7. Honeycomb Structures

**Figure 20:**
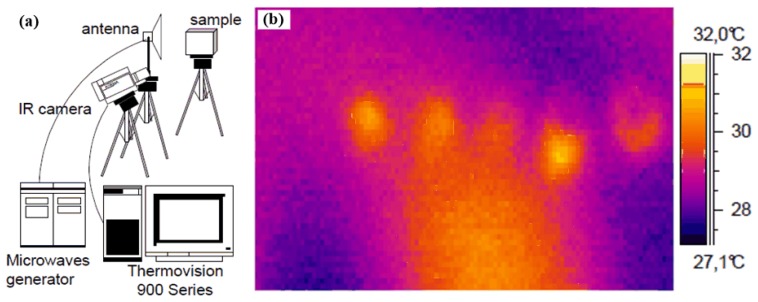
MWT setup at the Military Institute of Armament Technology (**a**) and results for water in the honeycomb material (**b**) [72].

Microwave pulsed thermography for water measurement in honeycomb materials was developed by scientists in Poland [72]. They introduced 2 GHz antennas with a 30° beam width. The surface of a honeycomb sample was illuminated with a 30 mW/cm^2^ power density. The antenna and IR camera were arranged in a reflection configuration. The proposed MWT detection system is shown in Figure 20a. The antenna was located at 1 m away from the sample under test and the IR camera was placed at 0.7 m. The sample was a 290 × 215 mm^2^ sandwich panel with two 0.7 mm thickness Fibredux face skins. Different quantities of water (5.0, 2.5, 1.2 and <1 mL) are introduced in the sample to form four defects. Microwaves were used to irradiate the sample for 5 s, and then the IR camera was used to capture thermal images for 20 s at a rate of 1 Hz. In their experiment results, as shown in Figure 20b, the location of water can be well visible even in small quantities with the reflection and transmission arrangement, but it is difficult to qualify precisely the water content due to the phenomenon of longitudinal heat diffusion.

### 4.8. Cement Based Composite

**Figure 21:**
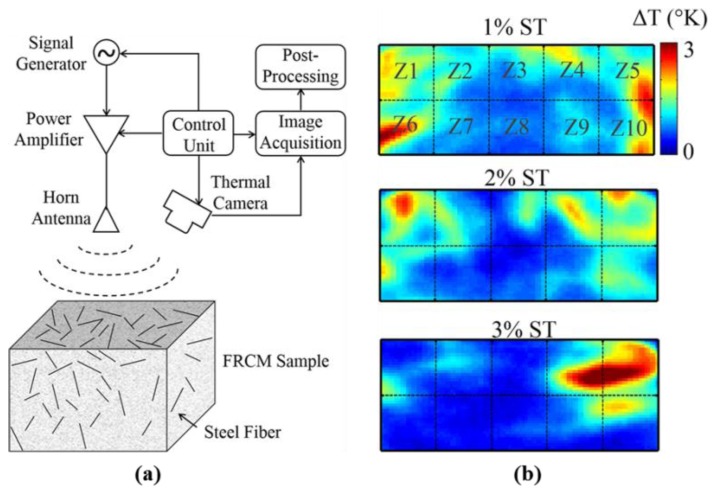
MWT measurement setup (**a**) and specimen surface temperature variation with different steel fiber contents (**b**) [73]. Reprinted/reproduced with permission from Springer.

Foudazi et al. proposed active MWT to evaluate steel fiber distribution in cement-based mortars [73]. 200 × 200 × 200 mm^3^ fiber-reinforced cement-based mortar (FRCM) samples were measured. The steel fibers have diameters of 0.55 mm and lengths of 30 mm. The effects of clumping, dielectric properties and fiber depth have been evaluated with a full-wave coupled electromagnetic-thermal simulation which was conducted by using CST MultiPhysics Studio^TM^. As illustrated in Figure 21a, microwave signals were generated by a signal generator at the desired 2.4 GHz operational frequency. A 50 W power amplifier was used to amplify the excitation power level. A horn antenna was used to radiate a relatively uniform microwave excitation toward the sample’s surface. A DRS Tamarisk 320 thermal camera was utilized to capture the surface thermal profile of the sample.

During measurements, microwave energy was applied for 30 s and an additional 90 s of thermal profile measurement followed to capture the cooling period. As shown in Figure 21b, surface temperature differences after 30 s microwave heating were provided for 1%, 2% and 3% of steel fiber contents. In their primary experiment results, they found that a larger temperature difference was contributed by the induced surface current on areas containing steel fibers. Therefore, with increasing volume content of steel fibers, the temperature will increase. Due to variation in heating associated with induced surface current and dielectric heating, fiber depth and dielectric properties of mortar have a significant influence on the temperature difference at the surface of samples. They found that MWT is capable of determining the presence of the fiber clumping in the cement-based composite structures: 1% and 2% steel fibers are shown to have higher surface temperature difference compared to the sample made with 3% fiber content.

### 4.9. Advanced Signal and Image Processing Methods

**Figure 22:**
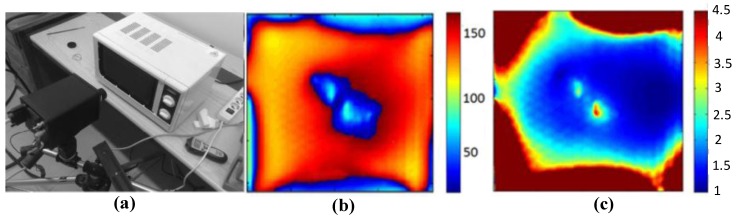
The experimental set-up used for relay (**a**), lock-in phase image (**b**) and amplitude image (**c**) [74].

Microwave lock-in thermography has been developed by scientists at Politecnico di Bari [74]. A function generator was used to control the power by switching the oven off/on. The frequency of excitation was 0.1 Hz. As shown in Figure 22a, the IR camera was positioned at 15 cm from the oven to allow it to focus on and frame the whole specimen. The dimensions of the specimen were 76 mm in length, 76 mm in width and 8 mm in thickness. Due to the low heat diffusion velocity and thermal conductivity, a strong drift in the temperature evolution over time is noted. Moreover, this setup can avoid damage due to possible microwave leakage. To obtain the amplitude and the phase information, Fast Fourier Transform-based algorithms were used to process the thermal image data which acquires thermal images frame by frame over time. Figure 22b shows the phase image and Figure 22c shows amplitude image. In the experimental results, the problem of the specimen’s edges was observed. Due to the interaction between the specimen geometry and the electromagnetic field, a high temperature was exhibited near the edge of the specimen. Nevertheless, the authors could clearly identify the damage area in the captured thermograms. In addition, quantitative analysis of the damaged areas showed a good agreement between the defect area results obtained with microwave thermography (1693 mm^2^) and X-ray scanning (1385 mm^2^).

**Figure 23:**
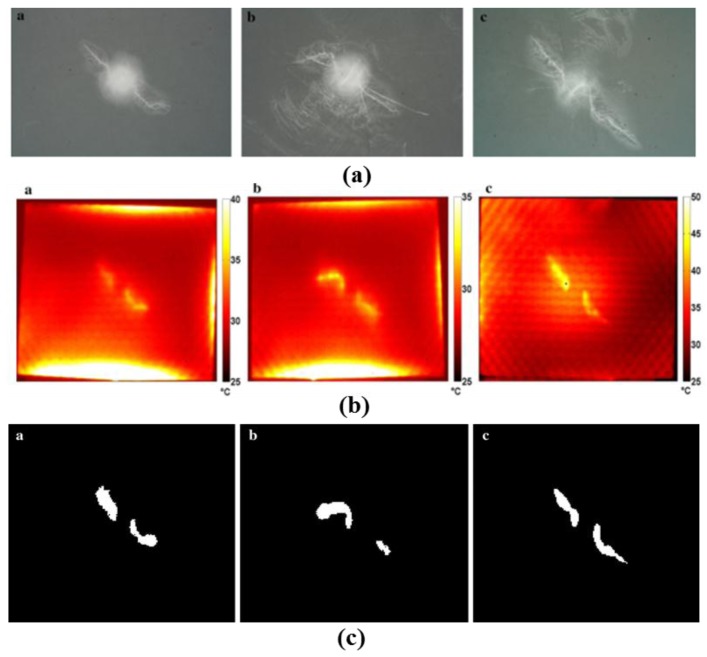
X-ray images of three samples (**a**), T_max_ maps during all acquisition sequence (**b**), and binary images obtained by T_max_ maps (**c**) [75]. Reprinted/reproduced with permission from Springer.

In 2014, Palumbo et al. investigated MWT for quantitative evaluation of damaged areas in CFRP through experiments and numerical simulations [75]. Damaged CFRP samples were inspected by microwave pulsed thermography. The dimensions of the four sandwich tested specimens were 76 × 76 mm^2^ with 8 mm thickness. Non-linear heating behavior was characterized and the undamaged area exhibited a higher slope during the heating and cooling phases. A new approach was proposed to process the obtained thermographic data. Numerical simulations were carried out to assess the sensitivity. An IR camera was located at 150 mm from the samples under test. To acquire the heating phase and subsequent cooling phase, the IR camera recorded for 7 s at 100 Hz. This proposed technique offers advantages in term of testing time (only 2 s of heating and a very fast data processing). In Figure 23a, X-ray images of the specimens under test are provided for comparison with the MWT results. As shown in Figure 23b, T_max_ analysis can be used to quantitatively indicate damaged areas located at a greater depth. The authors proposed a new algorithm based on the non-linear heating and cooling thermal behavior of damaged and undamaged areas for the quantification of damaged areas. The image after the data binarization is shown in Figure 23c. The location and size information of the damaged area can be obtained from these measured results. Quantitative analysis of the damaged areas shows a good agreement between results obtained with microwave thermography (784.3 mm^2^ with heating slope), X-ray (769.4 mm^2^) and lock-in thermography (779.3 mm^2^). The size of the minimum resolvable defect was 2 × 2 × 0.5 mm^3^, with a depth of 1.3 mm.

**Figure 24:**
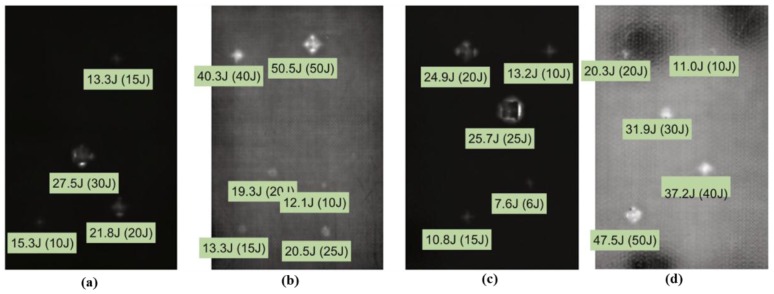
Estimated impact energy using artificial neural networks: specimen 2 front side (**a**), specimen 2 backside (**b**), specimen 3 front side (**c**), specimen 3 backside (**d**) [76]. Reprinted/reproduced with permission from Elsevier.

Usamentiaga et al. proposed an artificial neural network for automatic energy estimation of impact damages in carbon fiber composite materials, but the data acquisition time required for each inspection area was at least 30 s [76]. Three specimens were measured: specimen 1 was a carbon fiber composite made up of 12 plies with 2.5 mm in thickness. Specimens 2 and 3 were composite panels with two stiff facings made of carbon fiber and a low density material bonded between them. These two specimens have two valid sides, side A made up of six plies (1.5 mm in thickness) and side B made up of 12 plies (2.5 mm in thickness). A FLIR SC5000 IR camera was used to acquire infrared images for damages caused by impacts of different energy, ranging from 6 J to 50 J. With 12 bits per pixel, 320 × 256 pixel thermal images are produced by the FLIR SC5000 and its’ thermal sensitivity is 0.02 K. To cut down the number of obtained images, the experimental acquisition rate is 50 Hz while the maximum frame rate is 383 Hz. To improve the signal-to-noise ratio in the thermal images, a post-processing method was applied. With a Discrete Fourier Transform (DFT), the temperature-time history of each pixel during the heating period is transformed into the frequency domain and the phase information can be calculated based on it. As shown in Figure 24, the value below each defect is the estimated impact energy and the real impact energy is the number in brackets. In their primary experiment results, the impact energy of nearly half of the defects was estimated with an error of less than 2.5 J. This percentage increases to 80% when the considered error is 5 J, and nearly 100% when the estimated error is 10 J. The results indicate that an artificial neural network (ANN) is able to quantitatively characterize impact damages.

In summary, the purpose of adopting advanced signal processing methods in MWT is to eliminate noise, increase the signal to noise ratio for extracting the abnormality information of defects, enhance the contrast of detection images and improve the assessment accuracy. Meanwhile, the inspection time can be further reduced.

## 5. Discussion

### 5.1. Comparison with Other Excitation Sources

As mentioned before, a thermal contrast between surface/subsurface defects and the surrounding material can be produced by many energy sources. Some works are focused on the comparison between heating methods. For example, Keo et al. [56] compared microwave infrared thermography with CO_2_ laser thermography. MWT was found to be more suitable for the detection of deeper defects while CO_2_ laser thermography was more suitable for the detection of surface/near surface defects in CFRP.

According to a literature review [14,30,56,77], a comparison between MWT and other thermography excitation sources has been provided in Table 3. Due to the use of an IR camera, most of the thermography methods can offer fast inspection, non-contact, full-field, great sensitivity, high resolution, and quantitative analyses. As listed in Table 3, energy sources can be divided into:

1. Flash/lamp: halogen or IR lamps are commonly employed for a long period in large inspection areas with an x-y scanner/robot. In all cases, the measurement surface is been illumined by light to transfer heat and to propagate inside the specimen (containing a wavelength range from the ultraviolet, visible and infrared spectrum).
2. Laser: the heat is introduced into the material under test by the laser. Compared with lamps, a scan of the inspection area is needed.
3. Mechanical: sound or ultrasound waves are injected by transducers. With waves propagating through the specimen, heat is produced by slapping and rubbing of the surfaces (mostly in the defect areas). Compared with optical excitation, non-uniform heating is considerably reduced and the visibility of sub-surface defects is improved.
4. Induction: eddy currents are generated by an excitation coil. The penetration depth varies inversely with the operation frequency. The induction heating is limited to conductive materials. Compared with mechanical heating, heating non-uniformities have less influence in induction heating since heat is produced locally.
5. Microwave: the heat is introduced into the specimen by a time-gated microwave excitation source. The sub-surface microwave absorbing features can be used for measurements (such as metal bars/fibers, water-filled areas). By analyzing the time of the thermal images, quantitative defect information can be extracted (such as depth and size).

### 5.2. Comparison with Other NDT Methods

Eight major categories of NDT techniques are listed in Table 4. A comparison of these technologies is provided and an overview of each method given to identify the advantages and limitations of current NDT techniques.

For materials inspection, there is no universally applicable method. Selection of a particular NDT technique requires more consideration than the detection capabilities. Meanwhile, the application, portability of equipment, inspection schedule, inspection area, types of materials, accessibility, costs and expected defects types are also important.

### 5.3. Shortcomings of MWT

From the MWT literature, it can be found that MWT is not always perfect for quantitative material detection. There are still many shortcomings in existing studies, which can be mainly summarized as follows:

1. Inadequate theoretical studies

Multi-physics coupling mechanisms of metal/composite materials detection with microwave thermography are not deeply studied. For example, composite materials are typically composed of a variety of materials, and the microwave heating principles of composite materials are different from those of conductive and dielectric materials. Meanwhile, the physical processes of MWT for material evaluation are very complex, which includes microwave heating, heat conduction, and heat diffusion. For example, microwave heating represents a dielectric loss in glass fiber composite materials, where it is a volumetric heating method; while microwave heating is Joule heat for conductive materials which is affected by skin effects, and it is a surface heating method.

2. Lack of study on excitation signal modulation and corresponding data processing methods

As mentioned above, IR thermography techniques can be subdivided into pulse thermography, lock-in thermography, pulse phase thermography and step heating thermography [14]. Many scholars have studied microwave pulse thermography, microwave step heating thermography and microwave lock-in thermography. However, no researcher has studied pulse phase microwave heating in the frequency domain. Microwave pulse phase thermography combines the advantages of MPT and MLT which can inhibit change in the surface emissivity and other negative factors; the pulse width can bring a stronger contrast and a deeper defect can be detected.

3. Lack of systematic research on microwave excitation module optimization

Microwave excitation module is an important part of MWT, and the heating effect is directly reliant on it. Furthermore, the subsequent thermal imaging is directly affected by rapid and uniform heating. British and Polish researchers have investigated waveguides as excitation modules [57,72]; French scholars have studied the pyramidal horn antenna as an excitation module [56]; South Korean scholars have studied the coaxial setup as excitation module [69]. However, the advantages and disadvantages of these microwave excitation modules are not thoroughly studied, which results in is it being difficult to achieve the optimal detection ability with MWT systems.

4. Lack of internal properties characterization and defect quantification methods

Temperature variation from the infrared camera is a result of joint action by the surface properties of materials (emissivity), internal thermal properties (thermal conductivity, diffusivity, interlayer reflection coefficient), electrical properties (conductivity and permittivity) and other factors. How to extract these features from the surface temperature response and to further quantitatively characterize the material properties is important and difficult in the current studies. Existing studies did not provide an effective method for property characterization and defect quantification.

5. Lack of automatic separation and damage area quantification methods

Some scholars have studied several prefabricated macroscopic defects in composite materials (lamination defects, cracks and debonding, etc.), and experimental empirical formulas have been established; but there is a lack of related research on automatic separation of different defects and damage area quantification. The data acquired with MWT is an image sequence or a three-dimensional matrix. Matrix analysis method is theoretically possible to achieve fast imaging, automatic separation and damage area quantification. However, existing studies employed advanced matrix decomposition methods for MWT data processing.

## 6. Trends

1. Multiple physics and new physics

The physical properties of the specimens are different which results in the physics of MWT being different. For example, composites are multi-layered and their parameters are anisotropic as a result of fiber reinforcement. In addition, several composite materials are often included in a composite structure (such as a sandwich structure). Hence, the physics of metals differ from those of composites. Furthermore, the effects of the electromagnetic field, microwave propagation and other multiple-physical field also need to be investigated, such as thermal pattern interpretation [78] in thermal optical flow [79], and the spatial-, time-, frequency-, and sparse-pattern domains. Thus, multiple physics and new physics-based MWT methods are required for materials evaluation.

2. Computer simulation and modeling

Over the last several years, computer modeling and simulation (such as method of moments or MoM and finite element method or FEM) have been employed for understanding the physics during MWT measurements (such as microwave radiation and propagation, heat generation and diffusion). In the past, researchers have investigated three different approaches to resolving the electromagnetic phenomena of microwave propagation and heating processes. Firstly, time-domain solvers have been applied to microwave heating problems [80]. These use a time-marching algorithm to predict the electric and magnetic fields at the next time step. Secondly, frequency-domain methods have been investigated [81], where the numerical solution strategy uses a particular frequency to predict the electric and magnetic fields. Lastly, a method that combines an efficient time-domain solver with the power of a frequency-domain solver, has been used to predict the power distribution generated in a lossy medium during microwave heating [82]. Moreover, the operational frequency and radiation pattern of microwave excitation system can be optimized with simulation and modeling for better detection performance. In addition, the influence of materials’ properties (such as conductivity, dielectric, size and shape) can be investigated with simulation and the total cost of experiments will be reduced. What’s more, the parameters of defects (such as location, size, orientation and shape) can be examined. For composite materials, the influence of different fiber orientations in the microwave EM field can be investigated too. Therefore, simulation and modeling are needed to improve the reliability and accuracy of MWT systems.

3. Microwave excitation system optimization

MWT is based on microwave heating. The thermal profile of a material under test is created by an IR camera after microwave excitation. With MWT, a large amount of microwave excitation systems can be used to introduce the heat, however, the heating efficiency of microwave excitation systems is not only dependent on the properties of the microwave system (such as operational frequency, radiation pattern and power, etc.) but also relies on the physical properties of the material under test (such as size, shape, conductivity, dielectrics and microwave energy absorbing ability, etc.). Thus, the optimization of microwave excitation systems is required to improve the ability and sensitivity of MWT systems.

4. Signal processing algorithms

To extract useful features from the captured thermal images, advanced signal processing algorithms have been used. These algorithms includes wavelet transform [83], independent components analysis (ICA) [84], principal components analysis (PCA) [85,86], pattern recognition [87], support vector machine [88,89] and Tucker decomposition [90]. With suitable signal processing algorithms, the inspection results for size and depth identification, subsurface defect detection, emissivity variation reduction and defect dimension quantification can be significantly improved. Therefore, more advanced signal processing algorithms are needed to further improve the sensitivity and quantification ability of MWT systems.

5. Intelligent inspection systems

The efficiency of a MWT system can be improved by implementing an intelligent inspection system with artificial intelligence. As various types of defects can be acquired during material measurement, the treatment for different types of defects is different. Taking a composite material for example, the most common embedded defects are delamination, adhesive debonding and out-of-plane waviness. These defects are the most typical defects observed during manufacturing which need to be identified to improve the manufacturing quality of the composite. Therefore, it is important to classify the defect type with an intelligent inspection system. As computers become increasingly capable, artificial intelligence methods can be used in MWT to reduce the inspection time and improve the reliability of MWT systems. For example, Moomen et al. employed machine learning for feature selection in microwave NDT [91].

6. Mobile inspection systems

**Figure 25:**
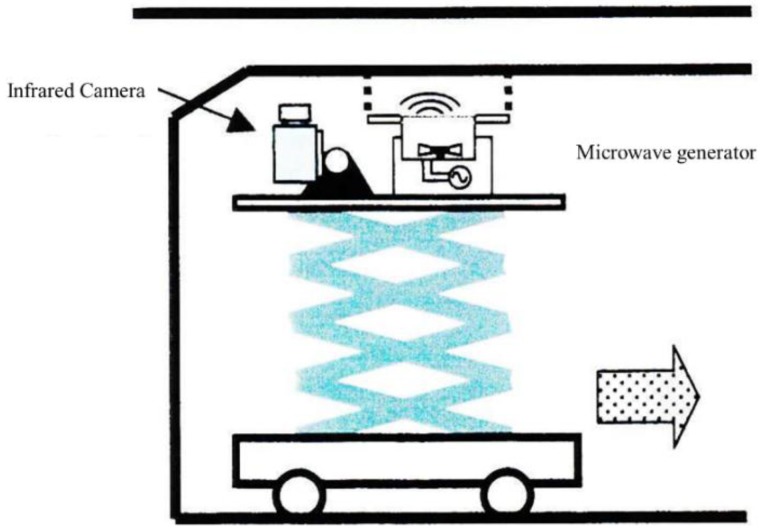
MWT combined with a vehicle [61]. Reprinted/reproduced with permission from SPIE.

For a large material under test, the MWT needs to be placed in a mobile robot or a vehicle. In Figure 25, a MWT inspection system has been combined with a vehicle [61]. The inspection time of MWT for a large material can be significantly reduced. The whole inspection can be performed autonomously. The safety and efficiency of the MWT systems are being improved too. However, lightweight equipment and advanced detection algorithms including compressed sensing are also required in order to provide automatic inspection capability.

## 7. Conclusions

The basic principles and types of MWT have been reviewed in this paper. MWT exhibits great potential, including fast heating, high resolution, fast inspection and high sensitivity, no contact requirement and better detectability for inner defects. Moreover, the manufacturing quality and reliability of materials can be improved to prevent failures. In this work, a comprehensive review of MWT techniques for material inspection has been reported based on a detailed literature survey. Firstly, the theory of MWT has been presented and MWT has been classified into four categories. Then, the development of MWT has been outlined through case studies. Next, limitations in current MWT research have been outlined based on detailed comparisons. Finally, some research trends in MWT are predicted. It is concluded that:

1. MWT combines the advantages of microwave technology and infrared thermography. A higher heating efficiency and uniform heating pattern can be expected. A full-field, non-contact, fast detection can be performed.
2. MWT can be divided into MPT, MPPT, MST and MLT. In the near future, microwave frequency modulated thermography and microwave pulsed phase thermography will be achieved.
3. MWT is a fast and effective non-destructive method for material inspection, especially for water/defects identification in concrete/composite structures.

## Figures and Tables

**Table 1:** Comparisons among MPT, MST, MLT and MPPT.

Techniques	Strength	Limitation
Microwave pulsed thermography [13]	Full-field, high resolution, high sensitivity, good visibility, quantification, fast, easy deployment	Small depth, long time for thick material, emissivity and non-uniform heating dependence, high power
Microwave step thermography [52]	Full-field, high resolution, high sensitivity, good visibility, quantification, fast, easy deployment, time-resolved	The radiation from heat source in the continuous heating process could have a negative influence on temperature measurements of MUT, emissivity, and non-uniform heating dependence
Microwave lock-in thermography [51]	Full-field, high resolution, higher sensitivity, good visibility, quantification, low power, emissivity independence, elimination of non-uniform heating	Compromise between depth and depth resolution, time-consuming,
Microwave pulsed phase thermography	Full-field, high resolution, high sensitivity, good visibility, quantification, fast, emissivity independence, elimination of non-uniform heating, greater depth and resolution, better detectability	Post signal processing, energy attenuation with frequency

**Table 2:** Summary of researches with MWT.

Hardware Development				Software Development			Experimental Study	
Operation Frequency	Antenna/Sensor	Power	IR Camera	Simulation Study	Sampling Software	Signal Processing	Material under Test	Defects
**8 GHz to 18 GHz [59]**	Horns and parabolic antennas [59]	100 W [59]	AGEMA 782 LWB [59]	Not available	CEDIP PTR-9010 system [59]	Normalized the infrared images pixel by pixel [59]	Glass-epoxy composites [59]	Delaminations
**2.45 GHz [60,61]**	Magnetron [60]	600 W–1700 W [60]	AGEMA AGA 880 [60]	Influence of defect permittivity and depth has been estimated [60].	HP 8757 C network analyzer [60]	Open cavity applicator and large cavity applicator have been designed [60]	Kevlar or fiberglass slabs and sandwich samples [60]	Defects
	Microwave oven [61]	1400 W [61]	Nikon LAIRD-3 [61]	Not available	Not available	Thermal image was taken at 20 s [61]	Mortar block [61]	Cracks
**2 GHz to 3 GHz [62], 2.45 GHz [63,64]**	TEM horn antenna [62,64]	50 W [62,64], 10 W [63]	DRS Tamarisk 320 [62,64], FLIR SC 500 [63]	CST Microwave Studio and MPHYSICS Studio [62].	Not available	The surface thermal profile was taken after 10 s of heating [62]. The surface thermal profile was taken after 5 s and 15 s of heating [63].	Reinforced steel bars [62], Rebar in air [63]; Embedded in cement [63,65], CFRP [64]	Corrosion, delaminations, debonding and crack
**2.45 GHz [65]**	Magnetron generated horn antenna [65]	600 W [65]	Not available	Not available	ALTAIR software	A contrast algorithm is used to analyze the thermogram series with 5 min of heating [65].		Steel bar corrosion
**5 GHz to 10 GHz [66]**	Horn antenna [66]	2.3 W [66,67]	Mikron 6T61 and SantaBarbara IR camera [66]	Not available	Macintosh microcompute [66]	A 2.7 s microwave pulse was used [66]	Multilayered plexiglass-water-teflon specimen [66]	Debonding
**9 GHz [67]**	A single flare horn antenna [67]		Santa Barbara Focalplane [67]	Not available	LabVIEW	The surface thermal profile was taken after 8 s and 10 s of heating [67]	Carbon fibers in different epoxy structures [67]	Embedded fibers
**18 GHz [57]**	Flann 18 094-SF40 waveguide [57]	1 W [57]	FLIR SC7500 [57]	COMSOL Multiphysics and CST Microwave Studio [57]	Rohde & Schwarz SMF 100 A signal generator [57]	The surface thermal profile was taken after 10 min of heating [57]	GFRP [57]	Defects
**2.45 GHz [56,68]**	WR430 waveguide [57]	1000 W [57]	Flir A325 [57,68]		Not available	The surface thermal profile was obtained after 10 s to 15 s of heating [57]		Debonding and delamination
	Magnetron [68]	500 W [68]		Not available		A sequence of 180 thermograms was obtained and processed by using normalized, standardized contrast and cosine transform [68]	Composite materials with adhesive bounded joints [68]	Defects
	Pyramidal horn antenna [56]	360 W [56]	Not available	Not available	ALTAIR program [56]	The surface thermal profile was taken after 150 s of heating [56]	CFRP [56]	Defects
**1 GHz [69]**	Coaxial-type probes [69]	0.1 W [69]	FLIR T620 [69]	CST Microwave Studio [69]	Spectrometer [69]	Normalized to the maximum value of field intensity [69]	Carbon-fiber composite materials [69]	Conductivity measurement

**Table 3:** Comparison of thermography inspection methods with different excitation sources.

Heating Sources	Strengths	Limitations
Flash, lamp	Non-contact, full-field, low cost, methods are mature	Surface heating, impact of surface condition on heating, heating reflection
Laser	Non-contact, remote heating from a far distance, high sensitivity, great resolution, quantification, fast	Heating area relies on excitation source, scanning is required, more suitable for surface defect detection
Mechanical	Full-field, high resolution, high sensitivity, quantification, fast, selective heating	Contact, know-how, specimen needs to be fixed, lack of quantitative information
Induction	Non-contact, relatively low-cost of excitation system, full-field, high resolution, great sensitivity, quantification, fast, inner heating	Limited to conductive material, non-uniform heating, complex heating system, near-field heating, heating area is limited to the excitation coil
Microwave	Non-contact, remote excitation, full-field, high resolution, great sensitivity, quantification, fast, uniform heating, selective heating	Complex and expensive microwave excitation system, electromagnetic radiation

**Table 4:** Summary and comparison for MWT with major NDT methods.

NDT Techniques	Strength	Limitation
**Ultrasound-echo/Phased array/Linear array**	Great depth, high resolution, many deployment options	Sound attenuation, coupling for contact testing, non-sensitive to surface defects
**Guide wave**	Large areas	Sound attenuation, coupling for contact testing
**Acoustic emission**	In-service, passive, large areas,	Noise, bad quantitation, non-sensitive to static defects
**Shearography**	Non-contact, full-field, fast, high sensitivity	Sensitive to part movement, small thickness/stiffness, require unique test set-ups, expensive, hard to quantitatively analyze
**Eddy current**	Non-contact, low-cost, no surface treatment	Conductive material, scanner required, sensitive to lift-off, low resolution
**Microwave**	Non-contact, high resolution, suitable for dielectric material	Scanner required, near-field, lift-off influence
**Microwave thermography**	Non-contact, full-field, high resolution, high sensitivity, quantification, fast, uniform heating, selective heating	Heating system complex, electromagnetic radiation
**X-ray/Gamma-ray**	High resolution, non-contact	X-ray radiation hazards, operation complex, scanner required

## Abbreviations

AHT	Abnormal heating thermography
ANN	Artificial neural networks
CFRP	Carbon fiber reinforced polymer
DFT	Discrete Fourier transform
ECLT	Eddy current lock-in thermography
ECPT	Eddy current pulsed thermography
ECPPT	Eddy current pulsed phase thermography
ECST	Eddy current step thermography
FEM	Finite element method
FRCM	Fiber-reinforced cement-based mortars
GFRP	Glass fiber reinforced polymer
LT	Lock-in thermography
MLT	Microwave lock-in thermography
MoM	Method of moments
MPT	Microwave pulsed thermography
MPPT	Microwave pulsed phase thermography
MST	Microwave step thermography
MT	Modulated thermography
MUT	Material under test
MWT	Microwave thermography
NDT	Nondestructive testing
PMC	Polymer matrix composites
PPT	Pulsed phase thermography
PT	Pulsed thermography
SHM	Structural health monitoring
SHT	Surface heating thermography
ST	Step thermography
VHT	Volume heating thermography
